# Surgery Is Not Associated With Early Presentation of Alzheimer's Disease and Mild Cognitive Impairment or Increased Risk for Both

**DOI:** 10.1002/cns.71149

**Published:** 2026-09-06

**Authors:** Wenzhe Zang, James Vithoulkas, Zhiyi Zuo

**Affiliations:** ^1^ Department of Anesthesiology University of Virginia Health Charlottesville Virginia USA

**Keywords:** age of diagnosis, Alzheimer's disease, mild cognitive impairment, surgery

## Abstract

**Background:**

Alzheimer's disease (AD) is the most common form of dementia in elderly patients. Mild cognitive impairment (MCI) may be a prodromal status of AD. Previous studies show inconsistent findings on whether surgery increases MCI and AD incidence. Moreover, it remains unknown whether surgery expedites the development of MCI or AD.

**Methods:**

This was a retrospective propensity score‐matched cohort study using data from the Alzheimer's Disease Neuroimaging Initiative (ADNI) database between September 2005 (ADNI initiation time) and May 2025 (time of data downloaded). Two outcomes were assessed: age at first diagnosis of MCI or AD, and incidence of MCI or AD. Nearest‐neighbor matching was used to balance demographic and comorbidity covariates.

**Results:**

The study was composed of 1114 subjects with MCI, 760 subjects with AD, and 397 subjects with normal cognition. Surgery was not associated with a decrease in the age of patients when MCI was diagnosed but was associated with a delayed presentation of AD by 1.7 years. Surgery performed on patients younger than 60 years was associated with a decrease in age when the first diagnosis of MCI or AD was made. Surgery at an age older than 60 years was associated with an increase in the age at MCI or AD diagnosis (mean difference: 5.1 and 5.6 years, respectively). Surgery was not associated with a change in the incidence of MCI or AD.

**Conclusion:**

Surgery may be associated with delayed presentation of AD or MCI, except for the surgery performed in patients younger than 60 years. Surgery may not affect the incidence of MCI or AD. These findings do not support deferring clinically indicated surgery for the concern of development of MCI or AD.

## Graphical abstract

Previous studies present inconsistent findings on the effects of surgery on the incidence of mild cognitive impairment (MCI) or Alzheimer's disease (AD). This study has shown that surgery is not associated with a change in the incidence of MCI and AD or expedition of their presentation.

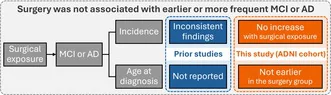

## 1 Introduction

Alzheimer's disease (AD) is a progressive neurodegenerative disorder characterized by the deposition of amyloid‐β plaques and intraneuronal neurofibrillary tangles composed of hyperphosphorylated tau in brain parenchyma and the cerebral vasculature [1, 2]. Surgery induces neuroinflammation [3, 4], a fundamental neuropathological process in the AD brain [2]. Animal studies have shown that hypothermia, hypoxia, and volatile anesthetics, conditions occurring and medications used during the perioperative period, may cause the deposition of amyloid‐β and hyperphosphorylated tau in mouse brain [5, 6, 7]. Additionally, there are significant increases of phospho‐tau and amyloid‐β in the cerebrospinal fluids after surgery [8, 9]. Thus, surgery may increase the risk for AD.

There are mixed conclusions on surgery as a risk factor for AD. A retrospective cohort study shows a higher incidence of AD in patients with surgery [10]. However, many other studies have found no association between surgery and AD. For example, no significant increase in the development of AD or other dementia is observed after appendectomy [11]. Patients with spinal surgery do not have a high risk for AD [12]. There is no association between surgery after 40 years of age and mild cognitive impairment (MCI). However, surgery after 60 years of age is associated with increased incident MCI, suggesting that older age at exposure may be an important factor to consider [13]. MCI is a clinical syndrome with multiple etiologies, including AD, cerebrovascular disease, neurodegenerative disorders other than AD, and non‐neurodegenerative conditions [14]. When accompanied by biomarker evidence of AD pathology, MCI represents an early symptomatic stage of AD rather than a distinct entity [15]. A recent study using the AD Neuroimaging Initiative (ADNI) database has shown that in general there is no association between surgery and MCI but surgery after age 75 and high‐risk surgeries are associated with greater risk for MCI [16]. ADNI is a longitudinal, multi‐center, observational database [17, 18].

Although many studies have explored whether surgery influences the risk of MCI or AD [10, 11, 12, 13, 18], few have adopted a case‐matching approach to control for confounding factors. Importantly, prior studies have not investigated whether surgery will expedite the development of MCI or AD. Therefore, we hypothesize that surgery is associated with the expedition of the development of MCI or AD. To address this hypothesis, we performed this retrospective propensity score‐matched study using the ADNI database.

## 2 Methods

This study was exempt from Institutional Review Board approval, given that it was a retrospective cohort analysis of a publicly available de‐identified database. For the same reason, informed consent from participants is not required.

### 2.1 Study Population

Data used in this article were obtained from the ADNI database (http://adni.loni.usc.edu) [17, 18]. ADNI is a multicenter, longitudinal observational study established to determine whether serial magnetic resonance imaging, positron emission tomography, chemical biomarkers, and clinical and neuropsychological assessments can be combined to measure the progression of MCI and early AD. Across successive phases (ADNI‐1, ADNI‐GO, ADNI‐2, ADNI‐3, and ADNI‐4), ADNI has enrolled volunteers aged 55 to 90 years at sites in the United States and Canada. Participants are recruited through participating memory and research clinics, community outreach, and referrals and are enrolled into prespecified diagnostic groups of normal cognition (NC), MCI, or AD. Each phase enrolls new participants and invites eligible participants from earlier phases to continue longitudinal follow‐up with clinical assessments, imaging, and biochemical assays.

Eligibility criteria were broadly consistent across ADNI phases. General inclusion criteria included: (1) having a study partner who had a minimal average of 10 h per week of contact with the participants and could accompany the participant to all study visits; (2) fluency in English or Spanish; (3) six or more grades of education or an equivalent work history; (4) visual and auditory acuity adequate for neuropsychological testing; (5) a modified Hachinski Ischemic Score of 4 or lower; (6) a Geriatric Depression Scale (15‐item short form) score below 6; (7) good general health with no diseases expected to interfere with the study; (8) stable doses of permitted medications for at least 4 weeks; and (9) willingness to undergo longitudinal neuroimaging, blood and cerebrospinal fluid sampling for genomic and biomarker analysis. Exclusion criteria included any significant neurological disease other than AD, an abnormal screening MRI, contraindications to MRI, major depression or bipolar disorder within the preceding year, a history of schizophrenia, alcohol or substance abuse or dependence within the preceding 2 years, significant systemic illness or unstable medical conditions, and residence in a skilled nursing facility. ADNI‐4 applied somewhat less restrictive criteria to broaden enrollment of underrepresented populations [17, 18].

In our study, 1874 ad or MCI participants and 397 participants with normal cognition (NC) who were enrolled between September 7, 2005 (the ADNI initiation time) and May 30, 2025 (the time of data downloaded) were included in the data analysis. Because ADNI enrolled selected participants rather than by sampling a defined population, the proportion of participants with MCI or AD in the database does not reflect the population prevalence of these illnesses.

### 2.2 Cognitive Function Assessment

The criteria for MCI diagnosis in the ADNI database were [15]: (1) mini‐mental state examination (MMSE) scores between 24 and 30 (inclusive); (2) subjective memory complaints reported by the patient, study partner, or clinician; (3) objective memory loss defined as scoring below an education‐adjusted cut‐off score on delayed recall of Story A of the Wechsler memory scale‐revised Logical Memory Test (score = 8 for those with ≥ 16 years of education; score = 4 for those with 8–15 years of education; score = 2 for those with 0–7 years of education); (4) a global clinical dementia rating (CDR) score of 0.5; and (5) absence of significant impairment in other cognitive domains, essentially preserved activities of daily living, and absence of dementia. These criteria selected amnestic MCI, which is commonly due to AD but can arise from other causes [14, 19].

The ADNI criteria for AD were [15]: (1) a global CDR score of 0.5 or 1.0; (2) MMSE scores between 20 and 26 (inclusive); (3) satisfying the National Institute of Neurological Disorders and Stroke‐Alzheimer's Disease and Related Dementias Association criteria for probable AD; and (4) other assessments based on neuropsychological testing, biomarkers, and neuropathology suggesting AD.

The criteria for normal cognition (NC) were: (1) MMSE scores between 24 and 30 (inclusive); (2) a CDR of zero; and (3) non‐depressed, non‐MCI, and non‐demented presentation.

Diagnoses and corresponding diagnosis dates were derived from the longitudinal study visits. After the initial screening and diagnosis, cognitive status was evaluated approximately every 6 to 12 months using the criteria above [15]. Due to the complicated disease progress nature of MCI and AD, 667 patients experienced varied diagnoses throughout the follow‐up visits. Among them, 547 participants underwent one change of diagnosis and 120 experienced two or more changes. The date on which a participant was diagnosed with AD for the first time was used as the diagnosis date of AD for this participant. The date on which a participant carried MCI diagnosis for the first time but had never been diagnosed with AD before the date was assigned as the diagnosis date of MCI for this participant. Of note, 417 participants who were diagnosed with MCI first and then AD contributed data to both MCI and AD groups. All participants in the NC group had normal cognition throughout the study visits.

### 2.3 Surgery Exposure, Outcomes, and Covariates

The primary independent variables were surgery, number of surgeries, and age at which surgery was performed. The dependent variables were age at which MCI or AD was diagnosed and the incidence of MCI or AD. Throughout this article, “incidence” denoted the proportion of participants carrying a diagnosis of MCI or AD in the cohorts of MCI and NC or AD and NC for each group defined by surgery exposure. Because the ratio of participants with and without cognitive impairment is determined by ADNI recruitment and no person‐time denominator in a true population is available, this proportion should be read as a relative comparison among different surgery exposure groups rather than as a population incidence rate.

Baseline characteristics included sex, body mass index (BMI), apolipoprotein E (APOE) ε4 genotype, self‐reported race and ethnicity, education level, marital status, functional status based on functional activities questionnaire (FAQ), hypertension, diabetes, endocrine metabolic disorder, respiratory disease, cardiovascular disease, renal‐genitourinary disease, current smoking, and dementia in mother or father. The age, BMI, APOE ε4 genotype, education level, and FAQ score are quantitative data while the other variables are categorical data.

Race was dichotomized into black and non‐black groups. Ethnicity was dichotomized into Hispanic/Latino and non‐Hispanic/Latino groups. Marital status was dichotomized to married and non‐married. The education level was the number of years in school. The FAQ score measured instrumental activities of daily living [20]. An integer score of APOE ε4 genotype (0, 1 or 2) was given to each patient: 0 = no alleles, 1 = one allele, 2 = two alleles. The age of surgery exposure was calculated by subtracting the year of surgery from the birth year. Of note, surgeries performed after the diagnosis date of MCI or AD were not considered because they would not affect the diagnosis age and incidence of MCI and AD.

### 2.4 Patient Inclusion and Exclusion Criteria

Participants from ADNI phases 1–3 were included in the analysis. Subjects in the ADNI Phase 4, which started patient enrolling in 2023, were not included due to the short follow‐up period. Those only with a diagnosis of significant memory concern were excluded. Patients with dementia of etiology other than AD were also excluded. Patients with early‐MCI and late‐MCI were categorized as MCI patients without distinction. Patients without the date of surgery were removed. Patients with incomplete information on demographic, clinical covariates, and missing follow‐up visits were also removed. For patients whose surgery dates included year but not month, June was assigned as the surgery month as an approximate average.

### 2.5 Statistical Analysis

To explore the impact of surgery on the development of MCI and AD, we analyzed the data in three perspectives: (1) whether the patients underwent any surgeries (i.e., impact of surgery in general), (2) the number of surgeries, and (3) the age at which surgery was performed. We determined the effects of each perspective on the diagnosis age and incidence of MCI and AD.

**FIGURE 1:**
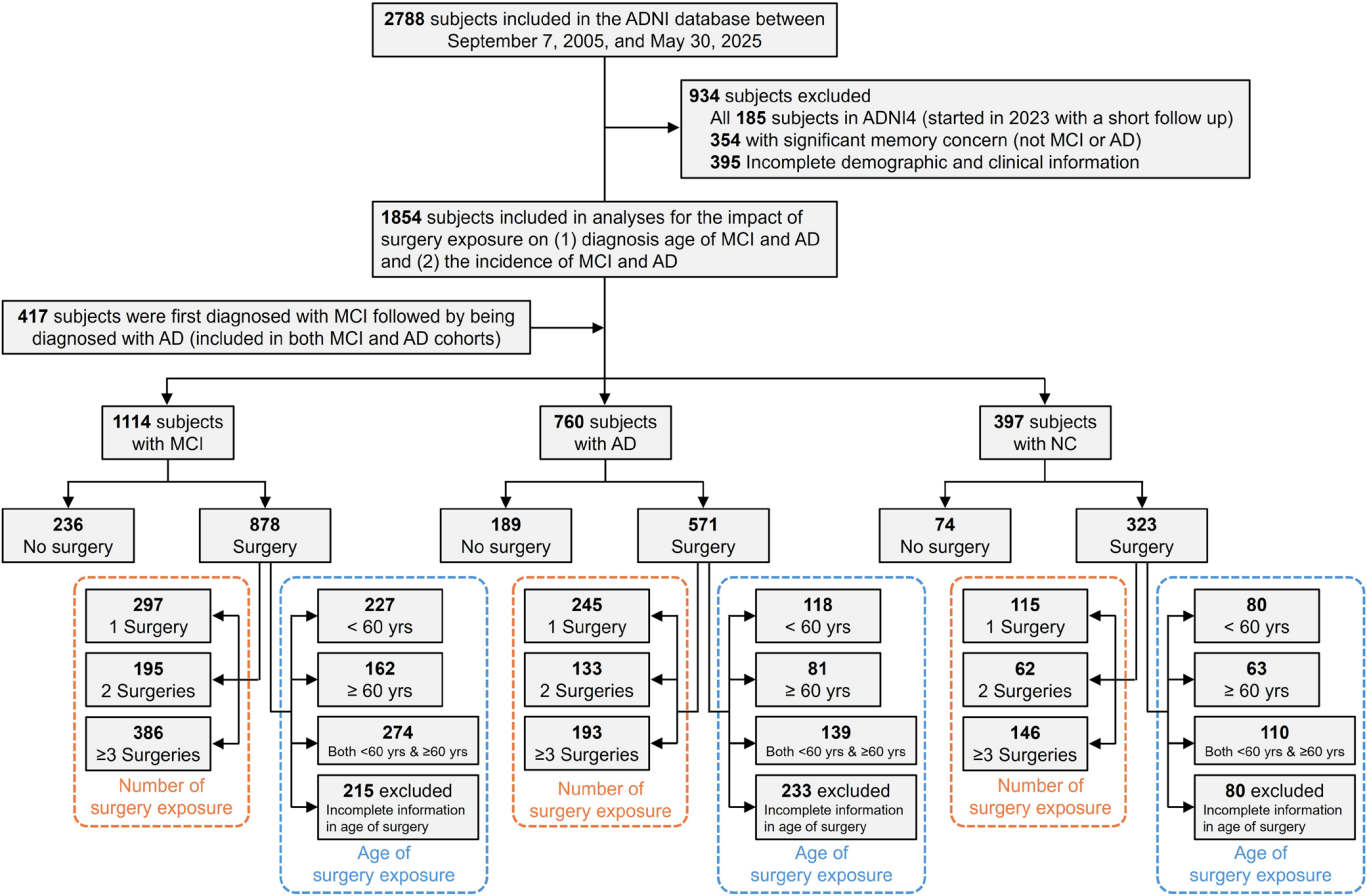
Flow diagram of study participants. Data of 2788 subjects were downloaded from the ADNI database spanning from September 7th, 2005, to May 30th, 2025. After excluding subjects who met the exclusion criteria, data of 1854 subjects were included in our final data analysis, of which 417 subjects were included in both MCI and AD groups because they were first diagnosed with MCI followed by AD. There were 1114 subjects with MCI, 760 subjects with AD, and 397 subjects with NC. Each group is then subdivided into surgery group (878 subjects with MCI, 571 subjects with AD, and 323 subjects with NC) and non‐surgery group (control group; 236 subjects with MCI, 189 subjects with AD, and 74 subjects with NC). Depending on the number of surgeries patients underwent, the surgery group is subdivided into 1, 2, and 3 or more surgery groups. Depending on the age when patients underwent surgeries, the surgery group is also subdivided into three groups: patients underwent surgeries at an age of younger than 60 years, 60 years or older, or both younger and older than or at 60 years.

When analyzing the impact of surgery in general, propensity score matching (PSM) was performed between non‐surgery and surgery groups based on patients' demographic and comorbidity information listed in Section 2.3. For the impact on diagnosis age, a 1:2 nearest neighbor PSM (NN PSM) algorithm using probit regression covariate estimates without replacement was applied to the MCI cohort and a 1:1 NN PSM algorithm was applied to the AD cohort. Regarding the impact on MCI and AD incidence, 1:2 NN PSM was performed for the MCI and NC cohorts and a 1:1 NN PSM was performed for the AD and NC cohorts. Balance among matched pairs was assessed by standardized mean differences. However, we did not use PSM when analyzing the effects of number or age of surgery exposure due to relatively small sample sizes in subgroups (Figure 1).

The associations between surgery, number or exposure age of surgeries, and the mean age to be diagnosed with MCI or AD or between surgery, number or exposure age of surgeries, and the incidence of MCI or AD were first assessed with univariate analyses. Student's *t*‐test was applied to determine whether surgery affected the ages when MCI or AD diagnosis was made. Both two‐sided and one‐sided (whether diagnosis age was increased or decreased) hypothesis tests were conducted. Chi‐squared (*χ* ^2^) test with continuity correction was used to determine whether surgery affected the incidence of MCI or AD. The data of participants without surgery was used as the reference. The statistical significance was defined by *α* ≤ 0.05.

Multivariable linear regression models were used to examine the impact of surgery, comorbidities, and demographic characters on the age to be diagnosed with MCI and AD. Multivariable logistic regression was used to determine the effects of surgery, comorbidities, and demographic characters on the incidence of MCI or AD. Results of these models are presented as *p* values, *β* coefficients, corresponding standard error, adjusted odds ratio (OR), and the corresponding 95% confidence intervals (CI).

Only complete cases were considered and missing data was not imputed during the analysis. Continuous quantitative variables were summarized by mean and standard deviation, while categorical variables were summarized by frequency (%).

The statistical analyses were performed by using R version 4.4.1 (R Core Team, Vienna, Austria) [21].

## 3 Results

### 3.1 Demographics and Sample Characteristics

**FIGURE 2:**
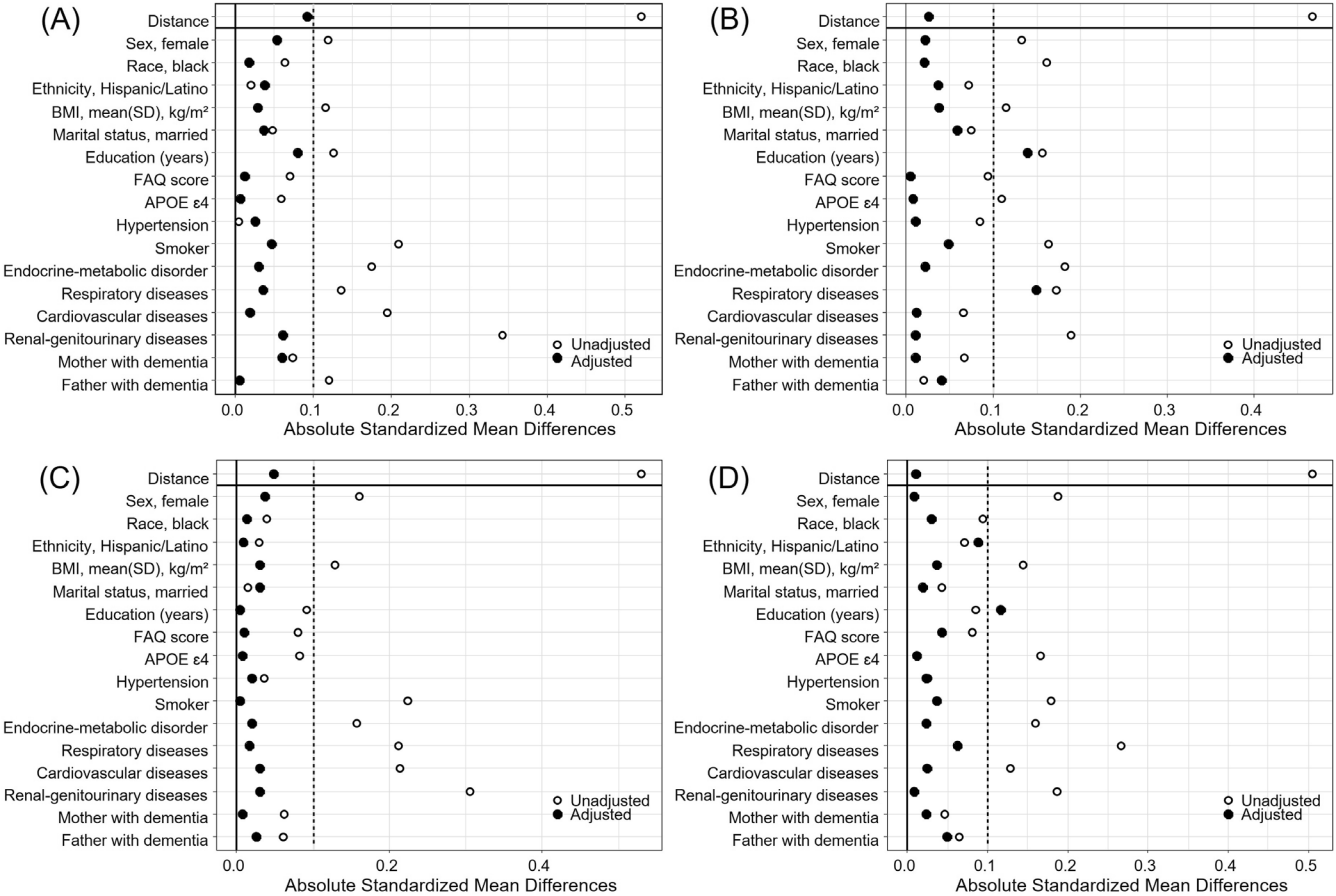
Love plot of propensity score matching results. (A) Absolute standardized mean differences before (*n* = 1114) and after (*n* = 708) matching in MCI cohorts with and without surgery exposure (2:1). (B) Absolute standardized mean differences before (*n* = 760) and after (*n* = 378) matching in AD cohorts with and without surgery exposure (1:1). (C) Absolute standardized mean differences before (*n* = 1511) and after (*n* = 930) matching in the combination of MCI and NC cohorts with and without surgery exposure (2:1). (D) Absolute standardized mean differences before (*n* = 1157) and after (*n* = 526) matching in the combination of AD and NC cohorts with and without surgery exposure (1:1). APOE ɛ4, Apolipoprotein E ɛ4 gene; BMI, body mass index; FAQ, functional activities questionnaire.

The flow diagram of the participant selection is presented in Figure 1. Among those participants included in the analysis, 1114 participants had MCI, 760 participants had AD, and 397 participants had normal cognition. Among them, 417 subjects were first diagnosed with MCI followed by a diagnosis of AD. These patients contributed data to both MCI and AD groups. Next, the cohorts of MCI, AD, and NC were sub‐divided into surgery and non‐surgery groups. Lastly, the surgery groups of MCI, AD, and NC cohorts were further divided based on the number and age of surgery exposure. The PSM results are presented in Figure 2 and Tables S1 and S2. This matching successfully reduced the difference in variables between patients with and without surgery or between patients with MCI or AD and normal cognition controls. Participants were followed for a median of 3.1 years (IQR 2.0 to 5.6), with the shortest individual follow‐up duration being 0.4 years and the longest 17.0 years, for a total of 8830 person‐years. The follow‐up of participants who were cognitively normal at baseline was longer (median 4.1 years, IQR 2.5 to 8.0 years).

### 3.2 Identification of Factors Affecting the Age at Which MCI or AD Was First Diagnosed

As shown in Table 1, surgery in general was not associated with a change in the age at which MCI was diagnosed. However, surgery delayed the first diagnosis of AD by 1.7 years.

**TABLE 1:** Univariate analyses (*t*‐test) of the effects of: (A) overall surgical exposure after propensity score matching, (B) number of surgeries, and (C) age of surgery exposure on the age when MCI or AD was diagnosed.

		Diagnostic age mean (SD)	Mean difference	*p*
				Two‐tailed, one‐tailed (increase), one‐tailed (decrease)
(A) Overall surgical exposure (propensity score‐matched)				
MCI	No	72.3 (7.6)	[Reference]	
	Yes	73.3 (7.7)	+1.0	0.110, 0.055, 0.945
AD	No	74.6 (7.7)	[Reference]	
	Yes	76.2 (7.9)	+1.7	0.037, 0.019, 0.981
(B) Number of surgeries				
MCI	0	72.3 (7.6)	[Reference]	
	1	73.7 (7.6)	+1.4	0.041, 0.021, 0.979
	2	73.4 (8.0)	+1.1	0.148, 0.074, 0.926
	≥ 3	74.4 (7.5)	+2.1	< 0.001, < 0.001, 1
AD	0	74.6 (7.7)	[Reference]	
	1	76.4 (8.1)	+1.8	0.018, 0.009, 0.991
	2	75.9 (7.6)	+1.3	0.132, 0.066, 0.934
	≥ 3	77.3 (7.6)	+2.7	< 0.001, < 0.001, 1
(C) Age of surgery exposure				
MCI	No	72.3 (7.6)	[Reference]	
	≤ 60	67.9 (7.6)	−4.4	< 0.001, 1, 0
	≥ 60	77.4 (6.0)	+5.1	< 0.001, 0, 1
	Both ≤ 60 and ≥ 60	75.3 (6.0)	+3.0	< 0.001, 0, 1
AD	No	74.6 (7.7)	[Reference]	
	≤ 60	72.0 (8.8)	−2.6	0.010, 0.995, 0.005
	≥ 60	80.1 (5.9)	+5.6	< 0.001, 0, 1
	Both ≤ 60 and ≥ 60	78.8 (6.6)	+4.2	< 0.001, 0, 1

*Note:* Part A is based on propensity score‐matched cohorts. Parts B and C are based on unmatched cohorts.

Findings from multiple linear regression analysis were consistent with the above univariate analysis results. There was no significant association between surgery and the age of developing MCI (*p* = 0.103). However, no surgery had a negative association with the age at which AD was diagnosed (*p* = 0.012), indicating that participants without surgery were diagnosed with AD at a younger age than those with surgery. Multivariate analysis identified that female gender, Hispanic/Latino, married status, and having APOE ɛ4 genotype, increased BMI or father with dementia reduced the age at which MCI was first diagnosed but having hypertension, cardiovascular diseases or renal‐genitourinary illness was associated with an increase in age at which MCI was diagnosed. Female sex, married status, Hispanic/Latino, having APOE ɛ4 genotype and increased BMI decreased the age to be diagnosed with AD but having renal‐genitourinary illness increased the age when AD was diagnosed (Table 2).

**TABLE 2:** Multiple linear regression with summarized statistics for the impact of overall surgical exposure on the age when MCI or AD was diagnosed.

Characteristics	Diagnosis age of MCI			Diagnosis age of AD		
	*p*	*β* coefficient (standard error)	Adjusted odds ratio (95% CI)	*p*	*β* coefficient (standard error)	Adjusted odds ratio (95% CI)
No surgery	0.103	−0.94 (0.57)	0.39 (0.13, 1.21)	0.012	−1.87 (0.74)	0.15 (0.04, 0.66)
Sex, Female	0.017	−1.48 (0.62)	0.23 (0.07, 0.76)	0.001	−2.73 (0.84)	0.07 (0.01, 0.34)
Race, Black	0.051	−2.42 (1.24)	0.09 (0.01, 1.01)	0.257	−1.79 (1.58)	0.17 (0.01, 3.67)
Ethnicity, Hispanic/Latino	0.002	−4.64 (1.52)	0.01 (0, 0.19)	0.002	−7.56 (2.44)	0 (0, 0.06)
BMI, mean (SD), kg/m^2^	< 0.001	−0.35 (0.06)	0.71 (0.62, 0.8)	< 0.001	−0.29 (0.09)	0.75 (0.63, 0.89)
Marital status, married	0.009	−1.94 (0.74)	0.14 (0.03, 0.61)	< 0.001	−4.65 (1.11)	0.01 (0, 0.08)
Education (years)	0.124	−0.16 (0.1)	0.86 (0.7, 1.04)	0.449	−0.12 (0.15)	0.89 (0.66, 1.2)
FAQ score	0.098	0.11 (0.07)	1.12 (0.98, 1.27)	0.817	0.01 (0.06)	1.01 (0.91, 1.14)
APOE ɛ4	< 0.001	−1.94 (0.42)	0.14 (0.06, 0.33)	< 0.001	−1.95 (0.54)	0.14 (0.05, 0.41)
Hypertension	0.003	2.07 (0.7)	7.94 (2, 31.49)	0.740	0.32 (0.97)	1.38 (0.21, 9.33)
Smoker	0.314	0.67 (0.66)	1.95 (0.53, 7.11)	0.614	0.44 (0.87)	1.55 (0.28, 8.45)
Endocrine‐metabolic disorder	0.366	−0.51 (0.56)	0.6 (0.2, 1.8)	0.998	0 (0.79)	1 (0.21, 4.7)
Respiratory diseases	0.479	−0.48 (0.67)	0.62 (0.17, 2.32)	0.663	0.41 (0.94)	1.51 (0.24, 9.45)
Cardiovascular diseases	0.031	1.6 (0.74)	4.95 (1.16, 21.12)	0.052	2.03 (1.04)	7.61 (0.99, 58.32)
Renal‐genitourinary diseases	0.007	1.55 (0.57)	4.71 (1.55, 14.31)	0.021	1.82 (0.78)	6.18 (1.33, 28.63)
Mother with dementia	0.053	−1.19 (0.61)	0.3 (0.09, 1.01)	0.070	−1.42 (0.78)	0.24 (0.05, 1.12)
Father with dementia	0.050	−1.22 (0.62)	0.3 (0.09, 1)	0.243	−1.12 (0.96)	0.33 (0.05, 2.13)

*Note:* Analyses are based on the propensity score‐matched cohorts.

Abbreviations: APOE ɛ4, Apolipoprotein E ɛ4 gene; BMI, body mass index; FAQ, functional activities questionnaire.

### 3.3 Effects of Numbers of Surgeries on the Age at Which MCI or AD Was Diagnosed

A single surgery and multiple surgeries were associated with an increase in the age of patients when MCI or AD was first diagnosed. The range of these increases was from 1.4 to 2.7 years. Two surgeries also tended to be associated with an increase in the age when MCI or AD was diagnosed but the difference did not reach statistical significance (Table 1).

### 3.4 Effects of Surgery Occurred at Various Ages on the Age When MCI or AD Was Diagnosed

Patients were divided into three groups depending on the ages when they had surgery: (1) before 60 years of age, (2) at or after 60 years of age, and (3) both before and at or after 60 years of age. Patients in the third group had at least 2 surgeries.

Univariate analysis (Table 1) showed that surgery performed on patients younger than 60 years was associated with a decrease in age when diagnosis of MCI (mean difference: 4.4 years) or AD (mean difference: 2.6 years) was made. Surgery at an age of ≥ 60 years was associated with an increase in the age when MCI or AD diagnosis was made (mean difference: 5.1 and 5.6 years, respectively). Patients with surgery both before and at or after 60 years of age had an increase in the age when MCI or AD was diagnosed (mean difference: 3.0 and 4.2 years, respectively).

### 3.5 Surgery Under Specific Circumstances Decreased the Incidence of AD

In the propensity score‐matched cohorts, surgery was not associated with a change in the incidence of MCI and AD. Three or more surgeries were associated with a decrease in the incidence of AD in the non‐propensity score‐matched cohorts (Table 3).

**TABLE 3:** Univariate analyses (*χ* ^2^ test) of the effects of (A) overall surgical exposure after propensity score matching, (B) number of surgeries, and (C) age of surgery exposure on the incidence of MCI or AD.

		Incidence MCI or AD/total (%)	Incidence difference	*p*
(A) Overall surgical exposure (propensity score‐matched)				
MCI	No	236/310 (76.1%)	[Reference]	
	Yes	453/620 (73.1%)	−3.1%	0.354
AD	No	189/263 (71.9%)	[Reference]	
	Yes	187/263 (71.1%)	−0.8%	0.923
(B) Number of surgeries				
MCI	0	236/310 (76.1%)	[Reference]	
	1	297/412 (72.1%)	−4.0%	0.255
	2	195/257 (75.9%)	−0.2%	1.000
	≥ 3	386/532 (72.6%)	−3.5%	0.291
AD	0	189/263 (71.9%)	[Reference]	
	1	245/360 (68.1%)	−3.8%	0.351
	2	133/195 (68.2%)	−3.7%	0.457
	≥ 3	193/339 (56.9%)	−15%	< 0.001
(C) Age of surgery exposure				
MCI	No	236/310 (76.1%)	[Reference]	
	≤ 60	227/307 (73.9%)	−2.2%	0.593
	≥ 60	162/225 (72.0%)	−4.1%	0.327
	Both ≤ 60 and ≥ 60	274/384 (71.4%)	−4.7%	0.183
AD	No	189/263 (71.9%)	[Reference]	
	≤ 60	118/198 (59.6%)	−12.3%	0.008
	≥ 60	81/144 (56.2%)	−15.7%	0.002
	Both ≤ 60 and ≥ 60	139/249 (55.8%)	−16.1%	< 0.001

*Note:* Part A is based on propensity score‐matched cohorts. Parts B and C are based on unmatched cohorts.

### 3.6 Identification of Factors Affecting the Incidence of MCI or AD

Consistent with the above univariate analysis results, surgery in general was not associated with a change in the incidence of MCI and AD. However, having APOE ɛ4 genotype and poor functional status were associated with an increase in the incidence of MCI and AD. Higher education was associated with a decrease in the incidence of MCI (Table 4).

**TABLE 4:** Multivariable logistic regression with summarized statistics for the impact of overall surgical exposure on the incidence of MCI or AD.

Characteristics	Incidence of MCI			Incidence of AD		
	*p*	*β* coefficient (standard error)	Adjusted odds ratio (95% CI)	*p*	*β* coefficient (standard error)	Adjusted odds ratio (95% CI)
No surgery	0.223	0.24 (0.2)	1.27 (0.87, 1.86)	0.620	0.55 (1.1)	1.72 (0.2, 14.86)
Sex, Female	0.090	−0.35 (0.2)	0.71 (0.48, 1.06)	0.887	−0.17 (1.17)	0.85 (0.09, 8.37)
Race, Black	0.717	−0.12 (0.33)	0.89 (0.47, 1.69)	0.638	−0.66 (1.41)	0.52 (0.03, 8.14)
Ethnicity, Hispanic/Latino	0.119	−0.69 (0.44)	0.5 (0.21, 1.19)	0.326	2.05 (2.08)	7.73 (0.13, 457.19)
BMI, mean (SD), kg/m^2^	0.355	−0.02 (0.02)	0.98 (0.94, 1.02)	0.367	0.1 (0.11)	1.11 (0.89, 1.38)
Marital status, married	0.239	−0.25 (0.21)	0.78 (0.52, 1.18)	0.878	−0.19 (1.25)	0.82 (0.07, 9.62)
Education (years)	0.038	−0.08 (0.04)	0.93 (0.86, 1)	0.063	−0.44 (0.24)	0.65 (0.41, 1.03)
FAQ score	< 0.001	1.73 (0.21)	5.64 (3.72, 8.56)	< 0.001	3.26 (0.79)	26.08 (5.52, 123.3)
APOE ɛ4	< 0.001	0.71 (0.16)	2.04 (1.49, 2.79)	0.003	3.4 (1.13)	30.09 (3.29, 274.74)
Hypertension	0.703	0.09 (0.25)	1.1 (0.68, 1.78)	0.513	0.96 (1.46)	2.6 (0.15, 45.66)
Smoker	0.647	0.11 (0.25)	1.12 (0.69, 1.82)	0.570	0.74 (1.31)	2.1 (0.16, 27.24)
Endocrine‐metabolic disorder	0.813	0.04 (0.19)	1.05 (0.73, 1.5)	0.375	−0.93 (1.05)	0.4 (0.05, 3.07)
Respiratory diseases	0.214	0.31 (0.25)	1.36 (0.84, 2.22)	0.565	−1.07 (1.87)	0.34 (0.01, 13.25)
Cardiovascular diseases	0.459	−0.19 (0.26)	0.83 (0.5, 1.37)	0.132	−2.37 (1.57)	0.09 (0, 2.04)
Renal‐genitourinary diseases	0.673	0.08 (0.2)	1.09 (0.74, 1.6)	0.424	−0.91 (1.13)	0.4 (0.04, 3.73)
Mother with dementia	0.241	−0.25 (0.21)	0.78 (0.52, 1.18)	0.904	−0.13 (1.08)	0.88 (0.11, 7.28)
Father with dementia	0.554	0.13 (0.21)	1.13 (0.75, 1.72)	0.055	−3.45 (1.8)	0.03 (0, 1.08)

Abbreviations: APOE ɛ4, Apolipoprotein E ɛ4 gene; BMI, body mass index; FAQ, functional activities questionnaire.

## 4 Discussion

Our results indicate that surgery in general is not associated with a change in the onset of MCI but may delay the presentation of AD. Surgery may not affect the incidence of AD or MCI. However, patients with 3 or more surgeries may have a delayed AD presentation and reduced AD incidence. These findings potentially provide assurance that patients receiving surgery do not face an early presentation and an increased risk of MCI or AD, and may encourage them to receive necessary surgeries. Possible reasons for this finding are that surgeries may improve patients' quality of life and functional status, and reduce chronic pain, which may be protective against AD [22]. An example of such a surgery may be arthroplasty for patients with severe osteoarthritis. Patients with osteoarthritis may have an increased risk of dementia [23, 24]. Chronic pain may play a role in this effect [23]. Arthroplasty may improve mobility and decrease pain, which may confer protection against dementia. Also, higher levels of physical activity may protect against AD and MCI [25, 26], which supports the hypothesis that improvements in functional status and mobility offer protection [27]. Also, an increased number of surgeries, while possibly indicative of chronic disease, may reflect greater access to care. Access to preventative care may confer protection against AD or MCI.

We chose to dichotomize the age at 60 years. This was an arbitrary decision, but the age of 60 years is often considered a separation between old and middle‐aged adults. Interestingly, patients with surgery before they were 60 years old had an early presentation of MCI or AD but surgery after that age delayed the presentation of MCI and AD. These novel findings suggest that age is an important factor for determining the effect of surgery on the development of MCI and AD. It is possible that surgery on younger patients may not provide as a big beneficial effect, such as reducing pain and improving physical activity, as for older patients because younger patients may be active and have less pain before the surgery. Also, it may take time for surgery to show the effect on the AD and MCI development. In supporting this possibility, a study has shown that surgery occurs within 20 years increases the risk for MCI but surgery within 5 years does not affect the risk for MCI [13]. However, a result that seems in conflict with this finding is that there is no increase in the risk for MCI in patients with surgery after they are 40 years old but there is an increased risk for MCI if surgery occurs after 60 years of age in the same study [13].

It is not surprising that female gender, Hispanic/Latino, APOE ɛ4 genotype, and increased BMI expedite the onset of MCI or AD. These factors are risk factors for AD [1, 28, 29, 30]. One significant finding is that married status is associated with a decrease in the age for the first diagnosis of MCI and AD. The reported effects of married status on the risk for AD or dementia are inconsistent [28]. Consistent with our finding, married status may increase the risk for AD [31]. Thus, married status may increase the incidence of AD and facilitate the presentation of AD. This possibility may be true or may be the result of living with a spouse who can detect abnormal cognition of the patient. Unmarried patients with MCI or AD can go unnoticed for a long time if they do not have frequent interactions with a relative and seek medical help. Thus, marital status may shift the age at which a diagnosis is recorded rather than the age at which the disease begins. One surprising finding is that patients with hypertension had a delayed presentation of MCI. Hypertension increases the risk for dementia [28]. Patients with untreated hypertension have increased risk for AD but patients whose hypertension is treated have a risk for AD similar to that of healthy control [32]. It is unclear how many patients have treated hypertension in our study. Another surprising finding is that cardiovascular diseases delayed the presentation of MCI. Cardiovascular diseases are risk factors for AD [28]. Similar to the issue of hypertension, it is unclear how many patients whose cardiovascular diseases are treated. Finally, patients with renal‐genitourinary issues may have a delay in the presentation of MCI and AD. Chronic kidney disease is thought to increase the risk for dementia [33]. However, renal diseases do not increase the risk for AD in two recent studies [34, 35]. Of note, it is not known whether hypertension and renal diseases increase the risk of MCI. Although hypertension, cardiovascular diseases, and renal‐genitourinary diseases are associated with a delay in the presentation of MCI or AD, they are not associated with a change in the incidence of AD or MCI in our cohorts.

Although the effects of surgery on the incidence of MCI or AD have been studied, many previous studies do not use a matching approach in the analysis [10, 13, 14]. We used PSM approach to derive two comparable groups. There was no difference in the incidence of MCI and AD between patients with and without surgery, which is consistent with studies that did control confounding by design [11, 12, 33, 34]. However, patients with three or more surgeries had a reduced AD incidence. The beneficial effects of surgery may be due to improved functional status and reduced pain. Poor functional status and having APOE ɛ4 genotype were associated with an increase in the incidence of MCI and AD. Having higher education levels reduced the incidence of MCI. These findings are consistent with the results reported in the literature [1, 28].

Inconsistent findings have been reported regarding the associations of anesthesia/surgery and AD. First, the biological effects of anesthesia and surgery may be acute and reversible rather than cumulative and prolonged. In animal models, anesthetics, hypothermia, and surgical stress increase tau phosphorylation, amyloid‐β generation, and neuroinflammation [3, 4, 5, 6, 7], and neuronal injury biomarkers rise transiently in human cerebrospinal fluid after surgery [8, 9]. However, surgery is associated with neither altered 1‐year cerebrospinal fluid amyloid‐β and tau trajectories nor greater amyloid burden on positron emission tomography [36, 37]. Second, many positive studies rely on administrative or claims data that lack information on cognitive status before surgery [10]. Cognitive dysfunction increases injury risk, such as fall and car accident, creating the possibility of reverse causation, in which perioperative health care contact leads to newly diagnosed cases of pre‐existing impairment. For example, one large cohort reported increased dementia risk after all types of anesthesia including regional anesthesia [10], a pattern that is difficult to reconcile with a specific anesthetic mechanism [38]. In contrast, studies that controlled confounding factors through a natural experiment [11] or prospective, protocol‐driven assessments [36, 37] found no association or that the effects are too small to be clinically meaningful.

Follow‐up duration in ADNI database varied among participants, with a median of 3.1 years (IQR 2.0 to 5.6 years). This variation could affect our findings, as participants with shorter follow‐up duration had less opportunity to convert from NC to MCI or from MCI to AD. Therefore, some eventual progressions may not have been observed, and the corresponding future converters may have been classified as NC or MCI. Because our analysis focused on differences between participants with and without surgery, this incomplete outcome ascertainment would be expected to affect both groups similarly and attenuate, rather than intensify, between‐group differences.

Our study possesses several strengths. First, we utilized the ADNI database, which recruits participants from multiple centers and adopted recognized diagnostic criteria for MCI and AD. Second, participants in the ADNI database are followed up every 6 to 12 months, which allows the time of MCI and AD diagnosis to be dated relatively accurately. This time may be close to the initial presentation of these illnesses. Third, this may be the first work exploring whether the age at which AD and MCI are first diagnosed is affected by surgery. Finally, PSM was performed to reduce the effects of confounding factors.

There are limitations in our study. First, this is a retrospective analysis. It is very difficult, if not impossible, to perform prospective and longitudinal studies to understand the risk for AD because of high cost and decades of follow‐up. In fact, all studies regarding the effects of surgery on AD development are retrospective [10, 11, 12, 13, 14]. Second, the exposure categories defined by age at which surgery are not structurally independent of the outcome. Participants classified as having surgery at or after 60 years of age are necessarily diagnosed with MCI or AD after that age; whereas participants without surgery are not. Thus, the associated difference in mean diagnosis age is partly arithmetic. This issue applies to the age‐of‐surgery analyses and does not affect the findings of the overall surgery exposure comparison. Third, although the ADNI database is large, some subgroups, such as AD or MCI patients without surgery, have a relatively small sample size, which prevents PSM in the analyses determining the effects of the number of surgery or age at which surgery was performed on the diagnosis of MCI or AD. Fourth, detailed surgical and anesthesia variables are not available in the database, which prevents us from grouping patients with the same type of surgery or anesthesia for comparison to reduce heterogeneity of the patients as in the postoperative cognitive impairment studies [11, 39, 40]. Fifth, although multiple clinical and demographic covariates were included in PSM, reliable indicators of socioeconomic status and access to medical care are not available for analysis. Sixth, the magnitude of the association should be interpreted with care. The FAQ measures instrumental activities of daily living, and impairment of daily function forms part of the diagnostic construct for dementia, while essentially preserved activities of daily living are part of the MCI diagnosis criteria (Section 2.2). The FAQ therefore overlaps the outcome definition rather than acting as a fully independent predictor, which may contribute to the large odds ratios in the MCI and AD models and to the wide confidence intervals seen across several covariates in the AD model. Finally, ADNI is a volunteer research cohort that is more educated and less ethnically diverse than the general population, which limits generalizability and constrains what can be inferred from the race and ethnicity covariates.

## 5 Conclusion

In summary, surgery in general is not associated with an early presentation of MCI but is associated with a delay in the presentation of AD. Patients with more than two surgeries may delay the presentation of MCI and AD by 2.1 and 2.7 years, respectively. Surgery before 60 years of age is associated with a reduction in the age of patients when MCI or AD is diagnosed. Surgery after 60 years of age is associated with an increase in the age when the diagnosis of MCI or AD is made. Finally, surgery is not associated with a change in the incidence of MCI and AD, and multiple surgeries may decrease the incidence of AD. These results suggest that surgery may delay the presentation of AD or MCI in specific conditions. These results do not support that surgery expedites the presentation of AD and MCI or increases their incidence and that patients need to cancel or delay the needed surgery due to the concern of risk for MCI or AD.

## Author Contributions

Z.Z. conceived the project; W.Z. and Z.Z. designed the studies; W.Z. downloaded the data from the ADNI database and performed the analysis with the input from Z.Z.; W.Z. drafted the manuscript; J.V. helped with the literature search and drafted part of the Introduction section; Z.Z. performed the final examination of the data analysis and revised the manuscript.

## Funding

This study was supported by the Robert M. Epstein Professorship Endowment (to Z.Z.), School of Medicine, University of Virginia, Charlottesville, VA, USA. The funder has no role in determining the study design, data analysis and interpretation, and publication.

## Disclosure

Raw data used in preparation of this article were obtained from the Alzheimer's disease Neuroimaging Initiative (ADNI) database (adni.loni.usc.edu). As such, the investigators within the ADNI contributed to the design and implementation of ADNI and/or provided data but did not participate in analysis or writing of this report. A complete listing of ADNI investigators can be found at: http://adni.loni.usc.edu/wp‐content/uploads/how_to_apply/ADNI_Acknowledgement_List.pdf.

## Ethics Statement

This study was exempt from Institutional Review Board approval, given that it was a retrospective cohort analysis of a publicly available de‐identified database. For the same reason, informed consent from participants is not required.

## Consent

The authors have nothing to report.

## Conflicts of Interest

The authors declare no conflicts of interest.

## Supporting information


**Table S1:** Demographics and medical comorbidities of the cohorts before and after propensity score matching: (A) MCI patients with or without surgery, and (B) AD patients with or without surgery.
**Table S2:** Demographics and medical comorbidities of the cohorts before and after propensity score matching: (A) MCI patients and NC participants with or without surgery and (B) AD patients and NC participants with or without surgery.

## Data Availability

The data that support the findings of this study are available from the corresponding author upon reasonable request.
