# Supplementary material for: Surgery Is Not Associated With Early Presentation of Alzheimer's Disease and Mild Cognitive Impairment or Increased Risk for Both
Source: CNS Neurosci Ther. 2026 Sep 6;32(9):e71149. doi: 10.1002/cns.71149 (PMC13547843; doi:10.1002/cns.71149)
Supplement: Supplementary file 1 [file CNS-32-e71149-s001.docx]

**Table S1.** Demographics and medical comorbidities of the cohorts before and after propensity score matching: **(A)** MCI patients with or without surgery, and **(B)** AD patients with or without surgery.

| (A) **MCI** | **Before propensity score matching, total n = 1114** | | | | | **After propensity score matching (2:1), total n = 708** | | | | | | |
| --- | --- | --- | --- | --- | --- | --- | --- | --- | --- | --- | --- | --- |
| **Characteristic** | **With surgery exposure (n = 878)** | **Without surgery exposure (n = 236)** | | | **P value** | **With surgery exposure (n = 472)** | | | **Without surgery exposure (n = 236)** | | | **P value** |
| Sex, Female | 370/878 (42.1%) | 86/236 (36.4%) | | | 0.114 | 184/472 (39.0%) | | | | 86/236 (36.4%) | | 0.511 |
| Race, black | 39/878 (4.4%) | 14/236 (5.9%) | | | 0.340 | 26/472 (5.5%) | | | | 14/236 (5.9%) | | 0.818 |
| Ethnicity, Hispanic/Latino | 29/878 (3.3%) | 7/236 (3.0%) | | | 0.795 | 17/472 (3.6%) | | | | 7/236 (3.0%) | | 0.660 |
| BMI (kg/m^2^) | 27.70 ± 5.01 | 26.57 ± 4.29 | | | 0.130 | 26.69 ± 4.83 | | | | 26.57 ± 4.29 | | 0.735 |
| Marital status, married | 675/878 (76.9%) | 186/236 (78.8%) | | | 0.529 | 379/472 (80.3%) | | | | 186/236 (78.8%) | | 0.643 |
| Education (years) | 15.89 ± 2.77 | 16.23 ± 2.74 | | | 0.089 | 16.01 ± 2.76 | | | | 16.23 ± 2.74 | | 0.315 |
| FAQ score | 2.94 (3.95) | 3.23 (4.16) | | | 0.334 | 3.28 ± 4.17 | | | | 3.23 ± 4.16 | | 0.878 |
| APOE ɛ4 | 0.57 ± 0.66 | 0.61 ± 0.66 | | | 0.425 | 0.61 ± 0.67 | | | | 0.61 ± 0.66 | | 0.936 |
| Hypertension | 441/878 (50.2%) | 118/236 (50.0%) | | | 0.950 | 242/472 (51.3%) | | | | 118/236 (50.0%) | | 0.750 |
| Smoker | 261/878 (29.7%) | 50/236 (21.2%) | | | 0.009 | 109/472 (23.1%) | | | | 50/236 (21.2%) | | 0.567 |
| Endocrine-metabolic disorder | 374/878 (42.6%) | 80/236 (33.9%) | | | 0.016 | 201/472 (42.6%) | | | | 97/236 (41.1%) | | 0.706 |
| Respiratory diseases | 247/878 (28.1%) | 53/236 (22.5%) | | | 0.081 | 99/472 (21.0%) | | | | 53/236 (22.5%) | | 0.753 |
| Cardiovascular diseases | 668/878 (76.1%) | 158/236 (66.9%) | | | 0.004 | 320/472 (67.8%) | | | | 158/236 (66.9%) | | 0.820 |
| Renal-genitourinary diseases | 477/878 (54.3%) | 89/236 (37.7%) | | | <0.001 | 192/472 (40.7%) | | | | 89/236 (37.7%) | | 0.447 |
| Mother with dementia | 305/878 (34.7%) | 74/236 (31.4%) | | | 0.330 | 135/472 (28.6%) | | | | 74/236 (31.4%) | | 0.449 |
| Father with dementia | 195/878 (22.2%) | 65/236 (27.5%) | | | 0.086 | 131/472 (27.8%) | | | | 65/236 (27.5%) | | 0.953 |
| (B) **AD** | **Before propensity score matching, total n = 760** | | | | | **After propensity score matching (1:1), total n= 378** | | | | | | |
| **Characteristic** | **With surgery exposure (n = 571)** | | **Without surgery exposure (n = 189)** | **P**  **value** | | **With surgery exposure (n = 189)** | | **Without surgery exposure (n = 189)** | | | **P**  **value** | |
| Sex, Female | 251/571 (44.0%) | | 71/189 (37.6%) | 0.123 | | 69/189 (36.5%) | 71/189 (37.6%) | | | | 0.831 | |
| Race, black | 16/571 (2.8%) | | 13/189 (6.9%) | 0.011 | | 12/189 (6.3%) | 13/189 (6.9%) | | | | 0.836 | |
| Ethnicity, Hispanic/Latino | 18/571 (3.2%) | | 4/189 (2.1%) | 0.462 | | 5/189 (2.6%) | 4/189 (2.1%) | | | | 0.736 | |
| BMI (kg/m^2^) | 26.43 ± 4.92 | | 25.91 ± 4.56 | 0.183 | | 26.08 ± 4.24 | 25.91 ± 4.56 | | | | 0.706 | |
| Marital status, married | 468/571 (82.0%) | | 160/189 (84.7%) | 0.397 | | 164/189 (86.8%) | 160/189 (84.7%) | | | | 0.557 | |
| Education (years) | 15.47 ± 2.91 | | 15.88 ± 2.62 | 0.070 | | 16.25 ± 2.65 | 15.88 ± 2.62 | | | | 0.179 | |
| FAQ score | 13.06 ± 6.53 | | 12.48 ± 6.29 | 0.273 | | 12.50 ± 6.82 | 12.48 ± 6.29 | | | | 0.969 | |
| APOE ɛ4 | 0.79 ± 0.71 | | 0.86 ± 0.66 | 0.201 | | 0.87 ± 0.76 | 0.86 ± 0.66 | | | | 0.942 | |
| Hypertension | 284/571 (49.7%) | | 102/189 (54.0%) | 0.313 | | 103/189 (54.5%) | 102/189 (54.0%) | | | | 0.918 | |
| Smoker | 189/571 (33.1%) | | 49/189 (25.9%) | 0.065 | | 53/189 (28.0%) | 49/189 (25.9%) | | | | 0.643 | |
| Endocrine-metabolic disorder | 257/571 (45.0%) | | 68/189 (36.0%) | 0.030 | | 81/189 (42.9%) | 79/189 (41.8%) | | | | 0.835 | |
| Respiratory diseases | 144/571 (25.2%) | | 35/189 (18.5%) | 0.060 | | 46/189 (24.3%) | 35/189 (18.5%) | | | | 0.168 | |
| Cardiovascular diseases | 419/571 (73.4%) | | 133/189 (70.4%) | 0.421 | | 132/189 (69.8%) | 133/189 (70.4%) | | | | 0.911 | |
| Renal-genitourinary diseases | 292/571 (51.1%) | | 79/189 (41.8%) | 0.026 | | 78/189 (41.3%) | 79/189 (41.8%) | | | | 0.917 | |
| Mother with dementia | 199/571 (34.9%) | | 72/189 (38.1%) | 0.420 | | 71/189 (37.6%) | 72/189 (38.1%) | | | | 0.916 | |
| Father with dementia | 110/571 (19.3%) | | 35/189 (18.5%) | 0.821 | | 38/189 (20.1%) | 35/189 (18.5%) | | | | 0.696 | |

Results are presented as mean ± SD or number of participants with a specific condition / total number of participants (%)

Abbreviations: BMI, body mass index; FAQ, functional activities questionnaire; APOE ɛ4, Apolipoprotein E ɛ4 gene.

**Table S2.** Demographics and medical comorbidities of the cohorts before and after propensity score matching: **(A)** MCI patients and NC participants with or without surgery and **(B)** AD patients and NC participants with or without surgery.

| (A) **MCI and NC** | **Before propensity score matching, total n = 1511** | | | **After propensity score matching (2:1), total n = 930** | | |
| --- | --- | --- | --- | --- | --- | --- |
| **Characteristic** | **With surgery exposure (n = 1201)** | **Without surgery exposure (n = 310)** | **P value** | **With surgery exposure (n = 620)** | **Without surgery exposure (n = 310)** | **P value** |
| Sex, Female | 551/1201 (45.9%) | 118/310 (38.1%) | 0.016 | 247/620 (39.8%) | 118/310 (38.1%) | 0.652 |
| Race, black | 66/1201 (5.5%) | 20/310 (6.5%) | 0.610 | 38/620 (6.1%) | 20/310 (6.5%) | 0.962 |
| Ethnicity, Hispanic/Latino | 49/1201 (4.1%) | 11/310 (3.5%) | 0.792 | 23/620 (3.7%) | 11/310 (3.5%) | 1.000 |
| BMI (kg/m^2^) | 27.17 ± 5.06 | 26.61 ± 4.34 | 0.052 | 26.49 ± 4.64 | 26.61 ± 4.34 | 0.677 |
| Marital status, married | 895/1201 (74.5%) | 233/310 (75.2%) | 0.875 | 458/620 (73.9%) | 233/310 (75.2%) | 0.730 |
| Education (years) | 16.03 ± 2.72 | 16.29 ± 2.76 | 0.150 | 16.30 ± 2.63 | 16.29 ± 2.76 | 0.959 |
| FAQ score | 2.17 ± 3.61 | 2.48 ± 3.87 | 0.204 | 2.45 ± 3.79 | 2.48 ± 3.87 | 0.895 |
| APOE ɛ4 | 0.49 ± 0.64 | 0.54 ± 0.64 | 0.196 | 0.54 ± 0.66 | 0.54 ± 0.64 | 0.914 |
| Hypertension | 591/1201 (49.2%) | 147/310 (47.4%) | 0.618 | 300/620 (48.4%) | 147/310 (47.4%) | 0.835 |
| Smoker | 343/1201 (28.6%) | 61/310 (19.7%) | 0.002 | 123/620 (19.8%) | 61/310 (19.7%) | 1.000 |
| Endocrine-metabolic disorder | 597/1201 (49.7%) | 130/310 (41.9%) | 0.017 | 254/620 (41.0%) | 130/310 (41.9%) | 0.832 |
| Respiratory diseases | 333/1201 (27.7%) | 60/310 (19.4%) | 0.003 | 124/620 (20.0%) | 60/310 (19.4%) | 0.884 |
| Cardiovascular diseases | 901/1201 (75.0%) | 201/310 (64.8%) | <0.001 | 411/620 (66.3%) | 201/310 (64.8%) | 0.714 |
| Renal-genitourinary diseases | 627/1201 (52.2%) | 116/310 (37.4%) | <0.001 | 241/620 (38.9%) | 116/310 (37.4%) | 0.721 |
| Mother with dementia | 406/1201 (33.8%) | 96/310 (31.0%) | 0.380 | 190/620 (30.6%) | 96/310 (31.0%) | 0.980 |
| Father with dementia | 274/1201 (22.8%) | 79/310 (25.5%) | 0.360 | 165/620 (26.6%) | 79/310 (25.5%) | 0.772 |
| (B) **AD and NC** | **Before propensity score matching, total n = 1157** | | | **After propensity score matching (1:1), total n = 526** | | |
| **Characteristic** | **With surgery exposure (n = 894)** | **Without surgery exposure (n = 263)** | **P value** | **With surgery exposure (n = 263)** | **Without surgery exposure (n = 263)** | **P value** |
| Sex, Female | 432/894 (48.3%) | 103/263 (39.2%) | 0.011 | 102/263 (38.8%) | 103/263 (39.2%) | 1.000 |
| Race, black | 43/894 (4.8%) | 19/263 (7.2%) | 0.170 | 17/263 (6.5%) | 19/263 (7.2%) | 0.863 |
| Ethnicity, Hispanic/Latino | 38/894 (4.3%) | 8/263 (3.0%) | 0.482 | 4/263 (1.5%) | 8/263 (3.0%) | 0.381 |
| BMI (kg/m^2^) | 26.80 ± 5.05 | 26.14 ± 4.55 | 0.046 | 26.31 ± 5.15 | 26.14 ± 4.55 | 0.698 |
| Marital status, married | 688/894 (77.0%) | 207/263 (78.7%) | 0.609 | 205/263 (77.9%) | 207/263 (78.7%) | 0.916 |
| Education (years) | 15.82 ± 2.82 | 16.05 ± 2.68 | 0.232 | 16.36 ± 2.75 | 16.05 ± 2.68 | 0.189 |
| FAQ score | 8.37 ± 8.13 | 8.99 ± 7.72 | 0.260 | 9.32 ± 7.93 | 8.99 ± 7.72 | 0.632 |
| APOE ɛ4 | 0.60 ± 0.69 | 0.71 ± 0.67 | 0.019 | 0.70 ± 0.72 | 0.71 ± 0.67 | 0.900 |
| Hypertension | 434/894 (48.5%) | 131/263 (49.8%) | 0.772 | 128/263 (48.7%) | 131/263 (49.8%) | 0.862 |
| Smoker | 271/894 (30.3%) | 60/263 (22.8%) | 0.022 | 64/263 (24.3%) | 60/263 (22.8%) | 0.758 |
| Endocrine-metabolic disorder | 451/894 (50.4%) | 112/263 (42.6%) | 0.030 | 109/263 (41.4%) | 112/263 (42.6%) | 0.860 |
| Respiratory diseases | 230/894 (25.7%) | 42/263 (16.0%) | 0.001 | 36/263 (13.7%) | 42/263 (16.0%) | 0.540 |
| Cardiovascular diseases | 652/894 (72.9%) | 176/263 (66.9%) | 0.069 | 179/263 (68.1%) | 176/263 (66.9%) | 0.852 |
| Renal-genitourinary diseases | 442/894 (49.4%) | 106/263 (40.3%) | 0.011 | 107/263 (40.7%) | 106/263 (40.3%) | 1.000 |
| Mother with dementia | 300/894 (33.6%) | 94/263 (35.7%) | 0.560 | 91/263 (34.6%) | 94/263 (35.7%) | 0.855 |
| Father with dementia | 189/894 (21.1%) | 49/263 (18.6%) | 0.425 | 54/263 (20.5%) | 49/263 (18.6%) | 0.660 |

Results are presented as mean ± SD or number of participants with a specific condition / total number of participants (%)

Abbreviations: BMI, body mass index; FAQ, functional activities questionnaire; APOE ɛ4, Apolipoprotein E ɛ4 gene.
